# A combined model for short-term wind speed forecasting based on empirical mode decomposition, feature selection, support vector regression and cross-validated lasso

**DOI:** 10.7717/peerj-cs.732

**Published:** 2021-09-24

**Authors:** Tao Wang

**Affiliations:** Hefei University of Technology, Hefei, China

**Keywords:** Wind speed forecasting, Empirical mode decomposition, Feature selection, Support vector regression, Cross-validated lasso, Multi-step wind speed forecasting

## Abstract

**Background:**

The planning and control of wind power production rely heavily on short-term wind speed forecasting. Due to the non-linearity and non-stationarity of wind, it is difficult to carry out accurate modeling and prediction through traditional wind speed forecasting models.

**Methods:**

In the paper, we combine empirical mode decomposition (EMD), feature selection (FS), support vector regression (SVR) and cross-validated lasso (LassoCV) to develop a new wind speed forecasting model, aiming to improve the prediction performance of wind speed. EMD is used to extract the intrinsic mode functions (IMFs) from the original wind speed time series to eliminate the non-stationarity in the time series. FS and SVR are combined to predict the high-frequency IMF obtained by EMD. LassoCV is used to complete the prediction of low-frequency IMF and trend.

**Results:**

Data collected from two wind stations in Michigan, USA are adopted to test the proposed combined model. Experimental results show that in multi-step wind speed forecasting, compared with the classic individual and traditional EMD-based combined models, the proposed model has better prediction performance.

**Conclusions:**

Through the proposed combined model, the wind speed forecast can be effectively improved.

## Introduction

As a sustainable and renewable energy alternative to traditional fossil fuels, wind power has attracted widespread attention and rapid development in recent years (Hu et al , 2018). According to the statistical report of the Global Wind Energy Council, the world capacity is about 650.8 GW (Fu et al., 2020), of which the installed capacity in 2019 is 59.7 GW (Global Wind Energy Council, 2020). However, with the increase of grid-connected wind power, the stability of the power system will be challenged (Liu et al., 2018a). This is because wind power is closely related to the non-stationarity of wind speed. Accurate wind speed forecasting will provide support for wind power planning and control, and even help reduce the impact of unexpected events on the stability of the power system (Liu et al., 2018b). But due to the non-linearity and non-stationarity of wind, it is difficult to establish a satisfactory wind speed forecasting model. To this end, researchers have made great efforts to improve forecasting performance from different aspects, including basic predictive models, preprocessing methods, and combined or hybrid strategies.

For basic predictive models, a variety of methods has been presented, mainly including physical models, statistical models, and machine learning. The physical model usually uses physical parameters such as temperature and pressure to predict wind speed (Heng et al., 2016). Numerical Weather Prediction (NWP) is one of the representative technologies. However, due to the weak correlation between physical parameters and short-term wind speed, this type of model can only be used for medium- and long-term wind speed forecasting, not for short-term wind speed forecasting. In the short-term wind speed forecasting, the wind speed is generally predicted by analyzing the inherent laws of historical wind speed data (Chen et al., 2018; Liu et al., 2018b).

The statistical model is a method widely used in short-term wind speed forecasting, which uses historical data to predict wind speed. Commonly used statistical models have autoregressive (AR) (Lydia et al., 2016a), autoregressive moving average (ARMA) (Torres et al., 2005) and autoregressive integrated moving average (ARIMA) (Wang & Hu, 2015). Kavasseri & Seetharaman (2009) proposed an *f*-ARIMA model for wind speed forecasting, and claimed that compared with the persistence model, their model has significantly improved the prediction accuracy. Ait Maatallah et al. (2015) developed a Hammerstein autoregressive model to predict wind speed, and verified that their model has a better root mean square error (RMSE) than ARIMA and ANN. Poggi et al. (2003) developed a model to predict wind speeds of three Mediterranean sites in Corsica based on AR, and proved that the synthetic time series can retain the statistical characteristics of wind speeds. Also, Lydia et al. (2016b) presented a short-term wind speed forecasting model by combining linear AR and non-linear AR. In general, the statistical model is based on the linear assumption of data, while the wind speed series have non-linear characteristics, which makes those methods unable to effectively deal with the non-linear characteristics of wind.

To solve the problem, machine learning is introduced by researchers to predict wind speed. Normally, machine learning is used as a predictive model or parameter optimization, mainly includes the evolutionary algorithm, extreme learning machine (ELM) algorithm, ANN algorithm and SVM algorithm. Wang (2017) presented a wind speed forecasting model by combining SVM and particle swarm optimization (PSO). Zhang et al. (2019) combined online sequential outlier robust ELM with hybrid mode decomposition (HMD) to predict wind speed. Wang, Li & Bai (2018) developed an error correction-based ELM model for short-term wind speed forecasting. Liu et al. (2020) introduced the Jaya-SVM (Jaya algorithm-based support vector machine) into wind speed forecasting. Krishnaveny et al. (Nair, Vanitha & Jisma, 2017) exploited the performance of three different models, *i.e.,* ANN, ARIMA and hybrid model, in wind speed forecasting. Azeem et al. (2018) investigated the KNN-based and ANN-based models for wind speed forecasting. Recently, deep learning, a new branch of machine learning, has received extensive attention. It has been widely used for regression and classification problems. According to the literature, deep learning can abstract the hidden structure and inherent characteristics of data compared with shallow methods. Khodayar & Wang (2019) introduced a scalable graph convolutional deep learning (GCDLA) for wind speed forecasting. Wang et al. (2016a) investigated a deep belief network model for wind speed forecasting. Khodayar & Wang (2019) combined rough set theory and restricted Boltzmann machines presented a wind speed forecasting. Hong & Satriani (2020) based on a convolutional neural network developed a day-ahead wind speed forecasting model. Although researchers claim that deep learning can achieve better performance, these methods are computationally intensive and prone to overfitting on small data sets.

In addition to these basic forecasting models, preprocessing methods such as feature selection (FS) are also introduced in wind speed forecasting. This is because in short-term wind speed forecasting, the lag of historical wind speed is usually used as the feature, which may lead to a certain degree of redundancy. FS is used to select the best input for the basic predictive model, so that the model can obtain better generalization performance (Li et al., 2018a). For example: Paramasivan & Lopez (2016) employed a ReliefF feature selection algorithm to identify key features, and then used a bagging neural network to predict the wind speed. Niu et al. (2018) presented a multi-step wind speed forecasting model using optimal FS, modified bat algorithm and cognition strategy. Botha & Walt (2017) combined FS with SVM to predict short-term wind speed. Kong et al. (2015) combined feature selection and reduced support vector machines (RSVM) for wind speed forecasting.

Due to the unstable nature of wind, the model of combined- or hybrid-signal processing technology has become the mainstream of wind speed forecasting. Wherein the signal processing technology is usually employed to decompose the wind speed to reduce or eliminate the instability. Commonly used signal processing techniques have empirical mode decomposition (EMD), variational mode decomposition (VMD) and wavelet transform (WT). Wang et al. (2016b) decomposed wind speed into stable signals using ensemble empirical mode decomposition (EEMD). Sun & Wang (2018) developed a fast ensemble empirical mode decomposition model to improve the accuracy of wind speed forecasting. Tascikaraoglu et al. (2016) based on WT proposed a wind speed forecasting model. Hu & Wang (2015) adopted an empirical wavelet transform (EWT) to extract key information in wind speed time series. Yu, Li & Zhang (2017) explored the performance of EMD, EEMD and complete ensemble empirical mode decomposition with adaptive noise (CEEMDAN) in wind speed forecasting.

In the field of wind speed forecasting, there are mainly three forecast scenarios: short-term forecasting, medium-term forecasting and long-term forecasting. Among them, short-term wind speed forecasting is essential for estimating power generation, and it is difficult to predict accurately due to the nonlinearity and instability of wind speed. Therefore, in the study, we tried to develop a new model to forecast short-term wind speed. The originality of this model is to propose a combined model of EMD, FS, SVR and Cross-validated Lasso (LassoCV) for multi-step wind speed forecasting. The framework of our study is as follows: (a) EMD is used to extract the intrinsic mode functions (IMFs) from the original wind speed time series; (b) FS and SVR are combined to predict high-frequency IMF; (c) LassoCV is used to complete the prediction of low-frequency IMF and trend.

The main contributions of the research are as follows:

1. A novel model based on EMD, FS, SVR and LassoCV is proposed to improve the accuracy of multi-step wind speed forecasting, where EMD is used to extract IMFs from the original wind speed data to reduce the non-stationarity of wind speed.
2. Based on the principle of EMD, the first IMF component decomposed by EMD contains most of the high-frequency information, and an algorithm with good generalization performance is usually required for prediction. We combine FS and SVR to predict the high-frequency IMF (*i.e.,* the first IMF) component.
3. Compared with the first IMF component, the frequency of the other IMF components decomposed by EMD is much lower and presents a Sin-like curve. Linear regression usually gets better performance. We introduce LassoCV to complete the prediction of low-frequency IMFs and trend.

The paper is as follows: The framework of the proposed model and the principles involved are introduced in ‘Methods’. ‘Results’ describes the experimental data used in the paper, and the comparison with the classic individual models. ‘Discussion’ discusses the effectiveness of EMD. ‘Conclusion’ concludes the study.

## Methods

### The whole process of the proposed model

**Figure 1:**
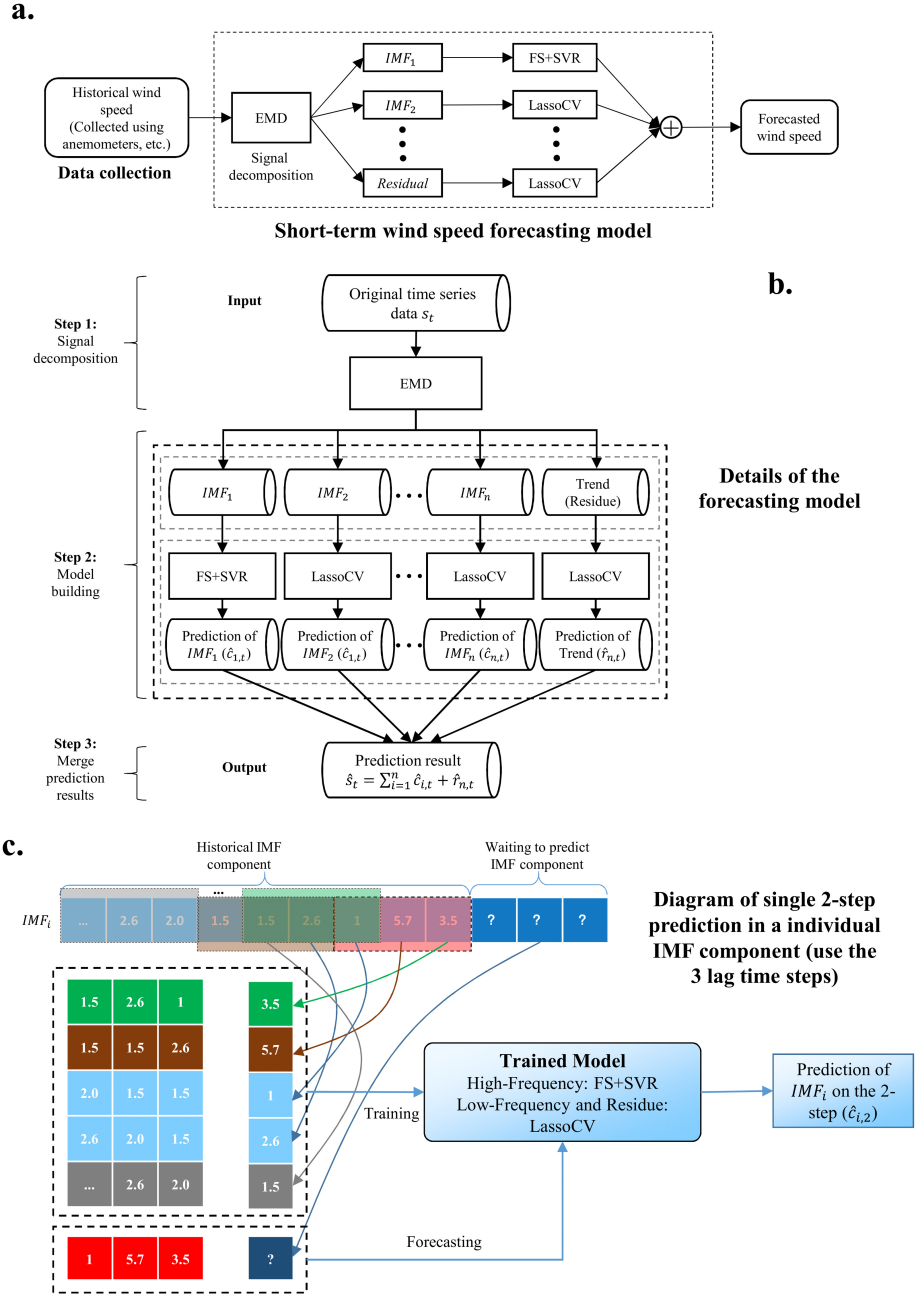
The whole process of the proposed model. (A) Short-term wind speed forecasting model. (B) Details of the forecasting model. (C) Diagram of single two-step prediction in an individual IMP component.

The architecture of our proposed model is shown in Fig. 1. The whole process is as follows:

1. Use EMD to decompose wind speed into a series of IMFs. EMD algorithm is introduced in ‘Empirical model decomposition’
2. Combine FS and SVR to predict the high-frequency IMF obtained by EMD. FS and SVR algorithms are provided in ‘Feature selection’ and ‘Support vector regression’, respectively.
3. Use LassoCV to complete the prediction of the low-frequency IMF and trend. LassoCV algorithm is listed in ‘Cross-validated lasso’.
4. Performance evaluation. The performance indicators are introduced in ‘Prediction performance criteria’, and the experimental results and analysis are given in ‘Results’ and ‘Discussion’.

### Empirical model decomposition

Due to the non-stationarity, intermittent and inherent nature of wind speed, it is difficult to directly predict the future wind speed. One possible solution is to decompose different frequencies from chaotic wind data (Bokde et al., 2019) and use models to predict them separately. Based on this idea, the study introduces signal processing technology to decompose wind speed. Common signal decomposition algorithms include Wavelet transform, morphology filters, EMD and many others. Wavelet transform is not adaptive and follows the prior knowledge of its mother wavelet, so somewhat limits its ability to extract nonlinear and non-stationary components from the data. Similarly, the morphology filters have to select the shape and the length of the structural element. There is no uniform standard and depends on human experience, whereas EMD has received great attention from researchers because of its superior performance and easy-to-understand. Therefore, in this study, we used EMD for preprocessing the wind speed.

EMD is essentially a non-linear signal analysis method that can handle non-linear and non-stationary time series (Huang et al., 1998). EMD uses the time-scale characteristics of the data to decompose the signal, and does not need to set any basis functions in advance. In theory, EMD can be applied to any type of signal. Since EMD was proposed, it has been rapidly applied to many different engineering fields such as marine and atmospheric research, seismic record analysis and mechanical fault diagnosis (Gao & Liu, 2021).

The basic idea of EMD is to decompose non-stationary time series signals into a series of IMFs along with a residue (Huang et al., 1998). The IMF should meet two principles: (1) the number of extreme and zero values must be equal or differ by at most one; (2) the average value of upper envelop and lower envelope must be zero (Ziqiang & Puthusserypady, 2007). Let $s \left( t \right) ,$t =1 , 2, …, *l* be a time series. EMD decomposition steps are as follows:

**Step 1:** Identify the local minima and maxima of the time series.

**Step 2:** Use cubic splines to interpolate local minima and maxima values to generate lower ${s}_{l} \left( t \right) $ and upper ${s}_{u} \left( t \right) $.

**Step 3:** Computer the average envelope of the upper and lower envelopes

\begin{eqnarray*}{m}_{t}= \frac{{s}_{u} \left( t \right) +{s}_{l} \left( t \right) }{2} \end{eqnarray*}

**Step 4:** Subtract the average envelope from the original time series $h \left( t \right) =s \left( t \right) -{m}_{t}$

**Step** **5:** Check $h \left( t \right) $ if meets the two principles of IMF. If so, treat $h \left( t \right) $ as the new IMF $c \left( t \right) $ and calculate the residual signal $r \left( t \right) =s \left( t \right) -h \left( t \right) $. Otherwise, replace $h \left( t \right) $ with $s \left( t \right) $, and then repeat steps 1 to 5.

**Step 6:** Set $r \left( t \right) $ as new $s \left( t \right) $ and repeat steps 1 to 5 until all IMFs are obtained.

Through the whole process, a set of IMFs from high to low frequency can be extracted from the time series. Therefore, the original time series can be expressed as:

\begin{eqnarray*}s \left( t \right) =\sum _{i=1}^{n}{c}_{i} \left( t \right) +{r}_{n} \left( t \right) \end{eqnarray*}

where *n* is the number of IMFs. ${c}_{i} \left( t \right) $ refers to the IMF, which is periodic and almost orthogonal to each other (Li et al., 2018b). ${r}_{n} \left( t \right) $ is the final residual representing the trend of $s \left( t \right) $.

### Feature selection

After obtaining the IMF components of wind speed, we need to predict it. In the study, we use the observed and lag of the IMF components as the raw features, respectively forecast each IMF component, and add all the predicted IMF components to get the final wind speed. Despite, the raw features contain sufficient information for forecasting, some irrelevant or partially relevant features in the raw features may have a negative impact on the model. To avoid the impact, a common strategy is to use feature selection to remove irrelevant features. Commonly used feature selection algorithms include filter method, wrapper method, heuristic search algorithm, embedded method (Chandrashekar & Sahin, 2014). In this study, we use the filter method. In order to obtain scores of different variables, we use the univariate linear regression test to calculate the correlation between features and output (Liu et al., 2019b), which is defined as:

\begin{eqnarray*}Co{r}_{i}= \frac{ \left( X \left[ :,i \right] -mean \left( X \left[ :,i \right] \right) \right) \mathrm{ \ast } \left( y-mean \left( y \right) \right) }{std \left( X \left[ :,i \right] \right) \mathrm{ \ast }std \left( y \right) } \end{eqnarray*}

where *X* is an *N* × *M* matrix, each column is a feature. *y* is the *N* × 1 vector of the output we are interested in. Based on the rank of correlation, the irrelevant or partially relevant features are removed.

### Support vector regression

The support vector machine (SVM) is a learning method based on structural risk minimization criteria, which can minimize the expected risk and obtain better generalization performance on unknown data. The support vector regression (SVR) is an extension of SVM for regression problems (Drucker et al., 1997). Due to the nonlinear and non-stationary nature of wind speed, SVR is widely used in short-term wind speed forecasting (Khosravi et al., 2018; Liu et al., 2019a; Santamaría-Bonfil, Reyes-Ballesteros & Gershenson, 2016). In the research, we use EMD to decompose the IMF components of wind speed, and the high-frequency IMF component contains the nonlinear and non-stationary part of wind speed. In order to obtain better generalization performance, we refer to existing research and use SVR to predict it.

The main idea of SVR is to implement linear regression in the high-dimensional feature space obtained by mapping the original input through a predefined function $\varnothing \left( x \right) $, and to minimize structure risks (Chen et al., 2018). Given a set of samples $ \left\{ {x}_{i},{y}_{i} \right\} ,$i =1 , 2, …, *N*, *y*_*i*_ is the output and *x*_*i*_ is the input. The objective is:

\begin{eqnarray*}\begin{array}{@{}c@{}} \displaystyle f \left( x \right) ={W}^{T}\varnothing \left( x \right) +b\\ \displaystyle R \left[ f \right] = \frac{1}{2} { \left\| W \right\| }^{2}+C\sum _{i=1}^{N}L \left( {x}_{i},{y}_{i},f \left( {x}_{i} \right) \right) \end{array} \end{eqnarray*}

where *W* and *b* are the regression coefficient and bias, respectively. *C* is the penalty coefficient. $L \left( {x}_{i},{y}_{i},f \left( {x}_{i} \right) \right) $ represents the loss function, and $R \left[ f \right] $ is the structure risk. The corresponding constrained optimization problem can be expressed as:

\begin{eqnarray*}\begin{array}{@{}c@{}} \displaystyle \mathit{min} \frac{1}{2} { \left\| W \right\| }^{2}+C\sum _{i=1}^{N} \left( {\xi }_{i}+{\xi }_{i}^{\mathrm{\ast }} \right) \\ \displaystyle \begin{array}{@{}cc@{}} \displaystyle s.t.&\displaystyle {y}_{i}-{W}^{T}\phi \left( x \right) -b\leq +{\xi }_{i}\\ \displaystyle &\displaystyle {W}^{T}\phi \left( x \right) +b-{y}_{i}\leq +{\xi }_{i}^{\mathrm{\ast }}\\ \displaystyle &\displaystyle {\xi }_{i},{\xi }_{i}^{\mathrm{\ast }}\geq 0,i=1,2,\ldots ,n \end{array} \end{array} \end{eqnarray*}

where *ξ*_*i*_ and ${\xi }_{i}^{\ast }$ refer to the slack variables. By introducing the Lagrange multiplier, the regression can be expressed as:

\begin{eqnarray*}f \left( x \right) =\sum _{i=1}^{N} \left( {\alpha }_{i}-{\alpha }_{i}^{\mathrm{\ast }} \right) K \left( {x}_{i},x \right) +b \end{eqnarray*}

where *α*_*i*_ and ${\alpha }_{i}^{\ast }$ are the Lagrange multipliers that satisfy the conditions ${\alpha }_{i}\geq 0,{\alpha }_{i}^{\ast }\geq 0$ and ${\mathop{\sum }\nolimits }_{i=1}^{N} \left( {\alpha }_{i}-{\alpha }_{i}^{\ast } \right) =0.K \left( {x}_{i},x \right) $ is the kernel function conforming to Mercer’s theorem.

### Cross-validated lasso

The Lasso algorithm is a regression model that can perform feature selection and regularization at the same time. It was originally proposed by Robert Tibshirani of Stanford University, with better prediction accuracy and interpretability (Tibshirani, 1996). Normally, in regression, we want to find a coefficient $\beta = \left( {\beta }_{1},\ldots ,{\beta }_{p} \right) $ that satisfies the following:

\begin{eqnarray*}Y=X\beta +,E \left[ {|}X \right] =0 \end{eqnarray*}

where *Y* is the dependent variable, $X= \left( {X}_{1},\ldots ,{X}_{N} \right) $ is the covariate, and *ɛ* is the unobserved noise. Lasso tries to minimize the objective function while forcing the sum of the absolute values of the coefficients to be less than a fixed value *t* (Hung, Yen & Li, 2016):

\begin{eqnarray*}{\min \nolimits }_{{\beta }_{0},\beta } \left\{ \frac{1}{N} \sum _{i=1}^{N}{ \left( {y}_{i}-{\beta }_{0}-{x}_{i}^{T}\beta \right) }^{2} \right\} \end{eqnarray*}

\begin{eqnarray*}s.t.\sum _{j=1}^{p} \left\vert {\beta }_{j} \right\vert \leq t. \end{eqnarray*}

Rewritten in the Lagrangian form:

\begin{eqnarray*}{\hat {\beta }}_{lasso}=\mathit{argmin}_{\beta \in {R}^{p}} \left\{ \frac{1}{N} { \left\| y-X\beta \right\| }_{2}^{2}+\lambda { \left\| \beta \right\| }_{1} \right\} \end{eqnarray*}

The *L*_1_-norm is used instead of the *L*_2_-norm in Lasso. Since the constraint region is diamond-shaped, it is more likely to pick the solution that lies at the corner of the region. As a result, the solution of the lasso is sparse, with some coefficients set to exactly equal to zero, that is, Lasso performs a straightforward feature selection.

To estimate ${\hat {\beta }}_{lasso}$, the value of the penalty parameter *λ* is critically important. However, the optimal *λ* is not given automatically. If *λ* is chosen appropriately, Lasso achieves the fast convergence under fairly general conditions; On the other hand (chosen inappropriately), Lasso may be inconsistent or have a slower convergence. In the paper, we adopt the cross-validated Lasso algorithm, in which the penalty parameter *λ* is chosen based on cross-validation, and this is also the leading recommendation way in the theoretical literature (Park & Casella, 2008).

### Prediction performance criteria

In the study the mean absolute percentage error (MAPE) , mean absolute error (MAE) and RMSE are used as performance indicators to evaluate the proposed wind forecasting model, which are defined as follows:

\begin{eqnarray*}MAPE& = \frac{1}{N} \sum _{i=1}^{N} \left\vert \left( {Y}_{i}-{\hat {Y}}_{i} \right) /{Y}_{i} \right\vert \end{eqnarray*}

\begin{eqnarray*}MAE& = \frac{1}{N} \sum _{i=1}^{N} \left\vert {Y}_{i}-{\hat {Y}}_{i} \right\vert \end{eqnarray*}

\begin{eqnarray*}RMSE& =\sqrt{ \frac{1}{N-1} \sum _{i=1}^{N}{ \left( {Y}_{i}-{\hat {Y}}_{i} \right) }^{2}} \end{eqnarray*}

where *Y*_*i*_ and ${\hat {Y}}_{i}$ refer to the observed and predicted wind speed of data point *i*, respectively. For MAPE, MAE, RMSE, the smaller value, the better the performance.

## Results

### Wind speed data

**Figure 2:**
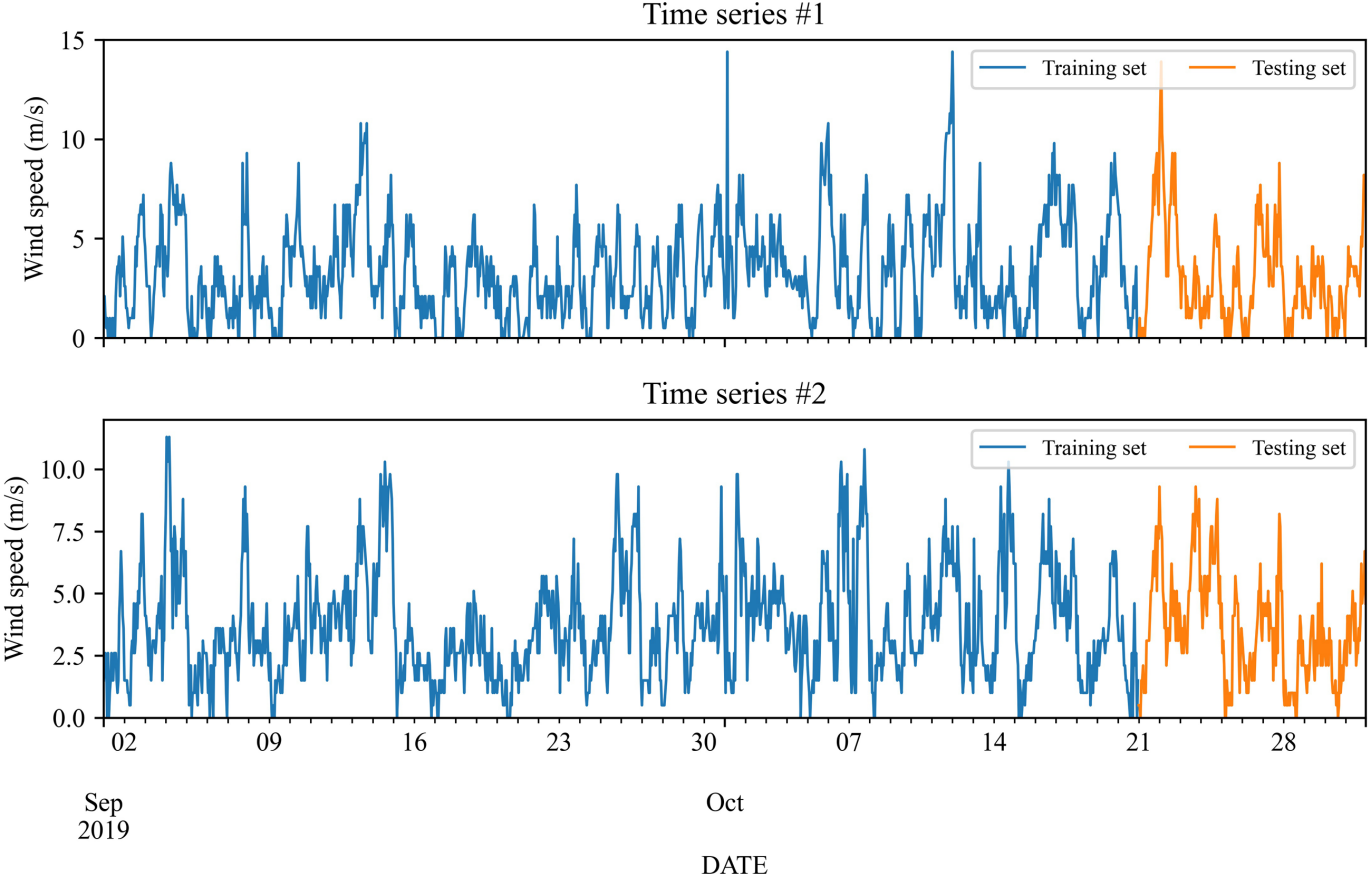
Wind speed collected from wind stations #1 and #2.

The wind speed data used in the study is gathered from two wind stations in Michigan, USA from September 2019 to October 2019. The number of data is 1,464. The initial 50 days from September 1, 2019 to October 20, 2019 are employed as input for model training, and the remaining days, *i.e.,* from October 21, 2019 to October 31, 2019 are used to test. Figure 2 shows these two wind speed time series, and the corresponding statistics are listed in Table 1.

**Table 1:** Wind speed statistics at wind stations #1 and #2.

**Wind station**	**Dataset**	**Date**	**Statistical indicators**					
			**Mean (m/s)**	**Max (m/s)**	**Min (m/s)**	**Std.**	**Stew.**	**Kurt.**
Site #1	Training set	Sept. 1, 2019 ∼ Oct. 20, 2019 (∼83%)	3.2975	14.4	0	2.378	0.871	0.865
	Testing set	Oct. 21, 2019 ∼ Oct. 31, 2019 (∼17%)	3.1614	13.9	0	2.486	1.108	1.312
Site #2	Training set	Sept. 1, 2019 ∼ Oct. 20, 2020 (∼83%)	3.6919	11.3	0	2.183	0.807	0.353
	Testing set	Oct. 21, 2019 ∼ Oct. 31, 2020 (∼17%)	3.5667	9.3	0	2.118	0.500	−0.318

### Experiments and result analysis

**Figure 3:**
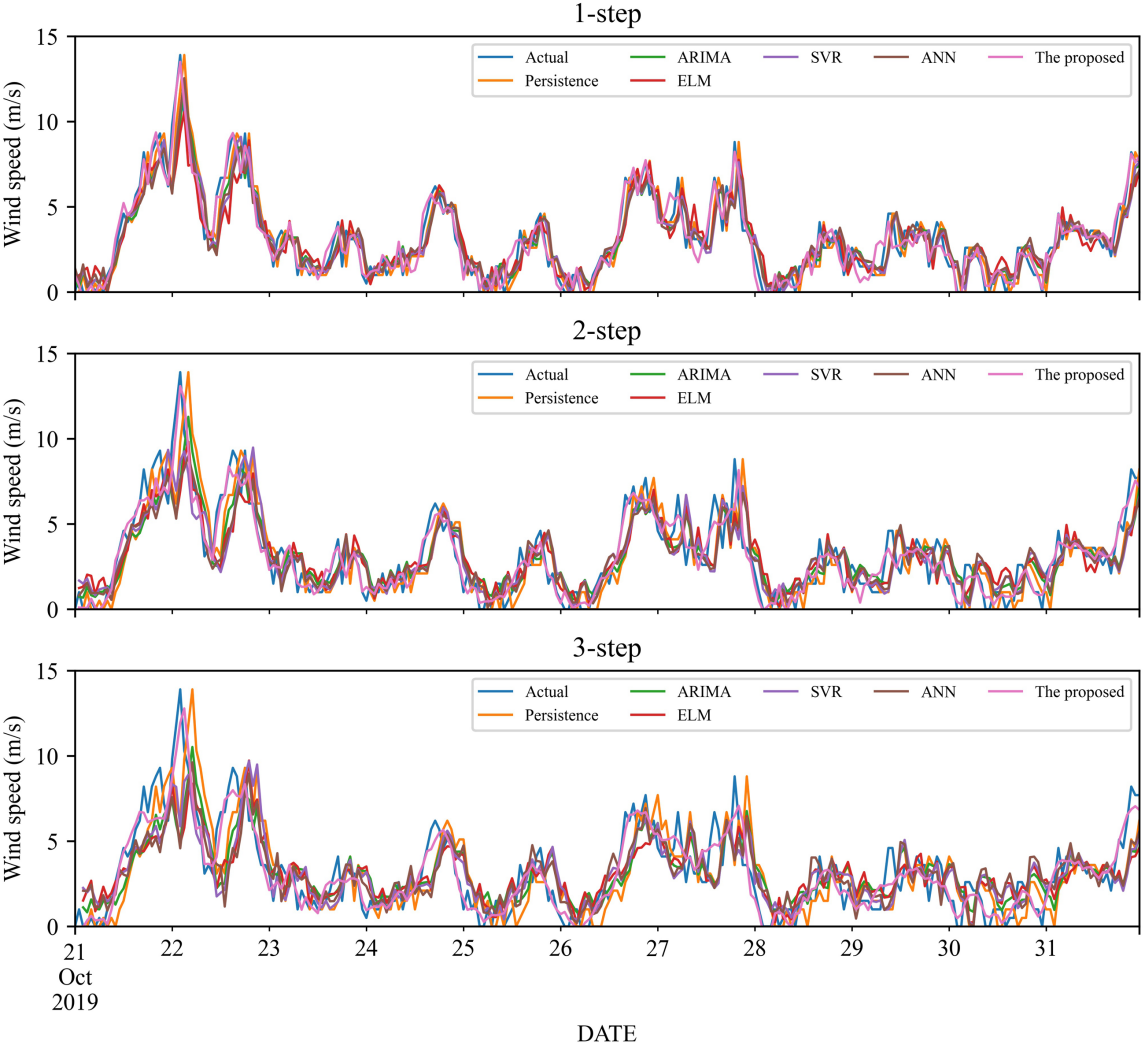
The prediction of the classic individual models at wind station #1.

**Figure 4:**
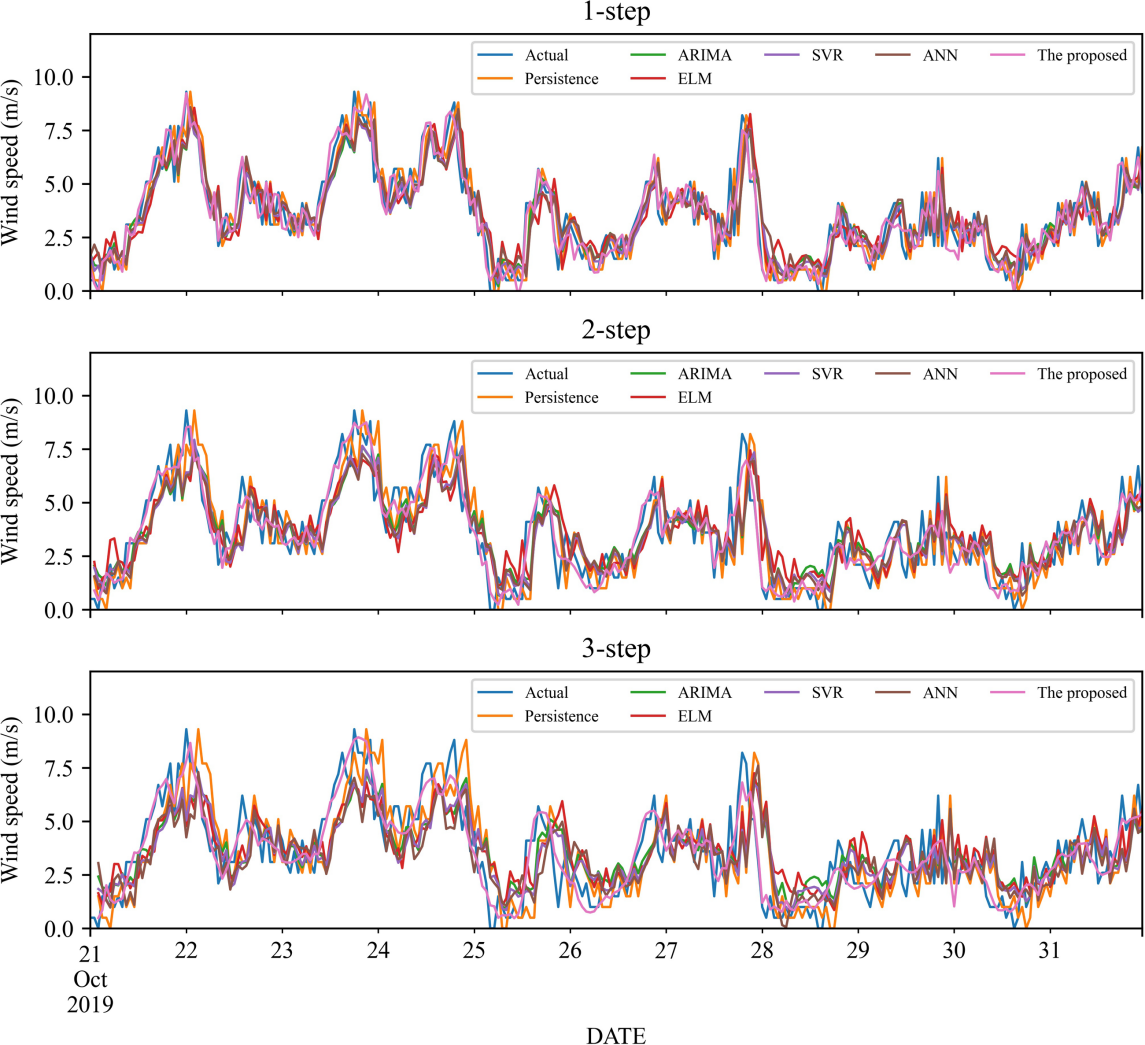
The prediction of the classic individual models at wind station #2.

To verify the effectiveness of the proposed model, we compare it with five classic individual models, including Persistence, ELM, SVR and ANN, ARIMA. The 1- to 3-step forecasting results of these models under time series #1 and #2 are displayed in Figs. 3–4, and the corresponding error estimated results are listed in Tables 2–5. It is worth noting that for a fair comparison, the parameters of the involved models are selected based on cross-validation. Based on the experimental results, we can get the following conclusions:

**Table 2:** The error result of the classic individual models at wind station #1.

**Models**	**1-step**			**2-step**			**3-step**		
	**RMSE**	**MAE**	**MAPE (%)**	**RMSE**	**MAE**	**MAPE (%)**	**RMSE**	**MAE**	**MAPE (%)**
Persistence	1.1892	0.8996	36.20	1.5892	1.2221	49.65	1.9008	1.4687	57.64
ARIMA	1.1724	0.9010	34.25	1.5182	1.1561	45.25	1.7647	1.3569	53.79
ELM	1.2705	0.9724	36.20	1.5500	1.1729	46.55	1.8109	1.3603	55.18
SVR	1.1739	0.9024	34.87	1.5676	1.1928	46.71	1.7832	1.3376	52.78
ANN	1.1984	0.9354	36.24	1.5338	1.1615	45.79	1.8427	1.3906	55.70
The proposed	0.5859	0.4426	21.11	0.7531	0.5848	24.78	0.8528	0.6798	27.55

**Table 3:** The error result of the classic individual models at wind station #2.

**Models**	**1-step**			**2-step**			**3-step**		
	**RMSE**	**MAE**	**MAPE (%)**	**RMSE**	**MAE**	**MAPE (%)**	**RMSE**	**MAE**	**MAPE (%)**
Persistence	1.2720	0.9739	35.98	1.4292	1.0947	41.02	1.6700	1.3073	47.99
ARIMA	1.1609	0.9302	38.71	1.3214	1.0430	45.43	1.5257	1.2188	53.05
ELM	1.2528	1.0188	44.81	1.3657	1.0915	51.40	1.5867	1.2849	60.12
SVR	1.1602	0.9218	36.51	1.3115	1.0360	43.63	1.5018	1.2008	49.91
ANN	1.1901	0.9460	40.62	1.3116	1.0330	43.72	1.6345	1.2798	52.18
The proposed	0.5593	0.4193	17.10	0.7540	0.5966	22.99	0.7911	0.6437	24.59

**Table 4:** The improvement rate of the proposed model relative to the classic individual models at wind station #1.

**Models**		**1-step**	**2-step**	**3-step**
Persistence	P_RMSE_ (%)	102.98	111.04	122.89
	P_MAE_ (%)	103.24	108.97	116.05
	P_MAPE_ (%)	71.47	100.34	109.26
ARIMA	P_RMSE_ (%)	100.11	101.60	106.94
	P_MAE_ (%)	103.55	97.69	99.60
	P_MAPE_ (%)	62.20	82.58	95.26
ELM	P_RMSE_ (%)	116.85	105.83	112.35
	P_MAE_ (%)	119.68	100.57	100.11
	P_MAPE_ (%)	71.44	87.82	100.31
SVR	P_RMSE_ (%)	100.36	108.16	109.11
	P_MAE_ (%)	103.87	103.96	96.76
	P_MAPE_ (%)	65.15	88.48	91.62
ANN	P_RMSE_ (%)	104.54	103.68	116.09
	P_MAE_ (%)	111.33	98.62	104.56
	P_MAPE_ (%)	71.63	84.78	102.23

**Table 5:** The improvement rate of the proposed model relative to the classic individual models at wind station #2.

**Models**		**1-step**	**2-step**	**3-step**
Persistence	P_RMSE_ (%)	127.42	89.54	111.11
	P_MAE_ (%)	132.24	83.49	103.08
	P_MAPE_ (%)	110.33	78.38	95.19
ARIMA	P_RMSE_ (%)	107.55	75.25	92.86
	P_MAE_ (%)	121.83	74.83	89.35
	P_MAPE_ (%)	126.31	97.56	115.77
ELM	P_RMSE_ (%)	123.99	81.12	100.59
	P_MAE_ (%)	142.95	82.96	99.60
	P_MAPE_ (%)	161.98	123.54	144.54
SVR	P_RMSE_ (%)	107.43	73.93	89.84
	P_MAE_ (%)	119.81	73.66	86.54
	P_MAPE_ (%)	113.43	89.76	103.00
ANN	P_RMSE_ (%)	112.78	73.95	106.62
	P_MAE_ (%)	125.58	73.16	98.82
	P_MAPE_ (%)	137.50	90.12	112.23

1. In the 1-step forecasting, for wind station #1, the proposed model obtains the best accuracy: RMSE, MAE, and MAPE are 0.5859, 0.4426, and 21.11%, respectively. The classic individual models from low to high based on RMSE are ELM, ANN, Persistence, SVR, and ARIMA, with MAPE values of 36.20%, 36.24%, 36.20%, 34.87%, and 34.25%, respectively. Likely, in wind station #2, compared with the classic individual models, the proposed model still obtains the best performance, and the MAPE value is 17.10%.
2. In the 2-step forecasting, when wind station #1 is used, the proposed model has the lowest performance criteria, *i.e.,* the values of RMSE, MAE, and MAPE are 0.7531, 0.5848, and 24.78%, respectively. In addition, for wind station #2, the proposed model still achieves the lowest performance criteria value. Take MAPE as an example, the value of MAPE is 22.99%, which is significantly lower than other models.
3. In the 3-step forecasting, the proposed model is still the model with the highest prediction accuracy, and the MAPE of wind stations #1 and #2 are 27.55% and 24.59%, respectively. Persistence has the worst RMSE value among these models, with MAPE of 57.64% and 47.99%, respectively.

In general, under 1- to 3-step forecasting, the proposed model can obtain the best prediction performance compared with the classic individual models.

### Compared with traditional EMD methods

**Figure 5:**
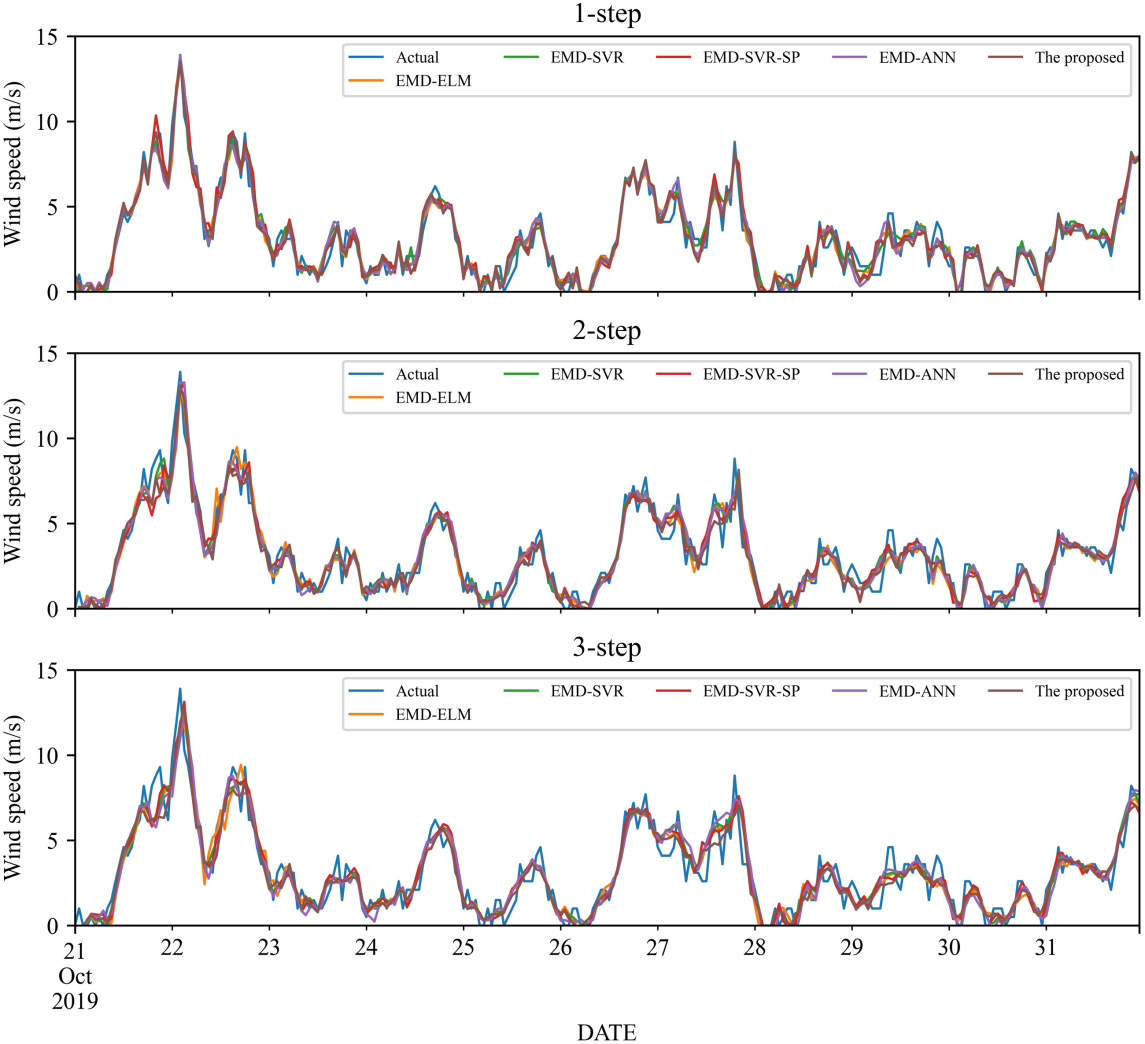
The prediction of different combination models at wind station #1.

**Figure 6:**
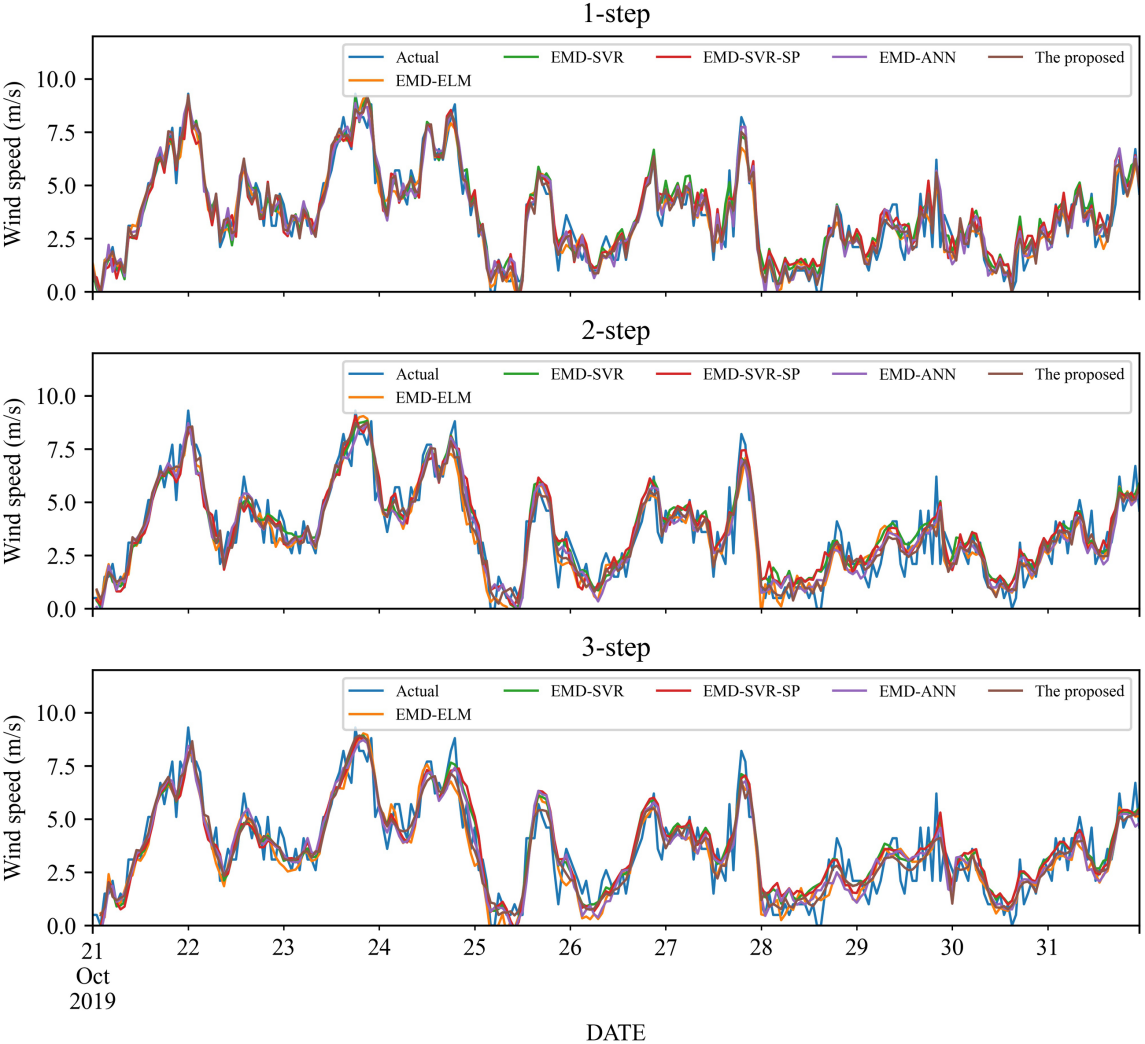
The prediction of different combination models at wind station #2.

As a nonlinear signal analysis method for processing nonlinear and non-stationary time series, EMD has been widely used in time series. To further verify the effectiveness of our EMD model, we compare it with four widely used EMD models, namely EMD-ELM, EMD-SVR, EMD-SP-SVR, and EMD-ANN. It is worth noting that in this study, these methods used the same way as our proposed model, using EMD to decompose the wind speed, using a single classifier to predict each IMF component separately, and adding all the prediction results to get the final prediction wind speed. The prediction results and the error estimated results of these four EMD-based methods and the proposed method are displayed in Figs. 5–6 and Tables 6–9. Based on Figs. 5–6 and Tables 6–9, it can be observed that:

**Table 6:** The error result of different combination models at wind station #1.

**Models**	**1-step**			**2-step**			**3-step**		
	**RMSE**	**MAE**	**MAPE (%)**	**RMSE**	**MAE**	**MAPE (%)**	**RMSE**	**MAE**	**MAPE (%)**
EMD-ELM	0.6400	0.5128	22.63	0.7854	0.6316	27.22	0.8746	0.6937	29.02
EMD-SVR	0.6379	0.5120	23.32	0.7768	0.6181	27.09	0.8583	0.6749	28.48
EMD-SVR-SP	0.6310	0.4867	23.03	0.7987	0.6141	26.30	0.8591	0.6762	28.66
EMD-ANN	0.6342	0.5055	23.55	0.7879	0.6221	27.67	0.8987	0.7040	29.31
The proposed	0.5859	0.4426	21.11	0.7531	0.5848	24.78	0.8528	0.6798	27.55

**Table 7:** The error result of different combination models at wind station #2.

**Models**	**1-step**			**2-step**			**3-step**		
	**RMSE**	**MAE**	**MAPE (%)**	**RMSE**	**MAE**	**MAPE (%)**	**RMSE**	**MAE**	**MAPE (%)**
EMD-ELM	0.6560	0.5283	21.59	0.8199	0.6669	27.49	0.8775	0.7096	27.65
EMD-SVR	0.6567	0.5233	24.88	0.8317	0.6736	29.85	0.8508	0.6986	30.52
EMD-SVR-SP	0.6437	0.4972	24.06	0.8211	0.6718	28.53	0.8894	0.7264	32.31
EMD-ANN	0.6397	0.5046	21.83	0.7927	0.6373	25.34	0.8520	0.6934	27.86
The proposed	0.5593	0.4193	17.10	0.7540	0.5966	22.99	0.7911	0.6437	24.59

**Table 8:** The improvement rate of the proposed model relative to other combined models at wind station #1.

**Models**		**1-step**	**2-step**	**3-step**
EMD-ELM	P_RMSE_ (%)	9.23	4.30	2.56
	P_MAE_ (%)	15.85	8.00	2.05
	P_MAPE_ (%)	7.20	9.82	5.36
EMD-SVR	P_RMSE_ (%)	8.88	3.16	0.65
	P_MAE_ (%)	15.67	5.69	−0.72
	P_MAPE_ (%)	10.43	9.31	3.41
EMD-SVR-SP	P_RMSE_ (%)	7.70	6.06	0.74
	P_MAE_ (%)	9.95	5.01	−0.53
	P_MAPE_ (%)	9.09	6.10	4.05
EMD-ANN	P_RMSE_ (%)	8.25	4.63	5.39
	P_MAE_ (%)	14.21	6.38	3.56
	P_MAPE_ (%)	11.52	11.67	6.40

**Table 9:** The improvement rate of the proposed model relative to other combined models at wind station #2.

**Models**		**1-step**	**2-step**	**3-step**
EMD-ELM	P_RMSE_ (%)	17.29	8.74	10.93
	P_MAE_ (%)	25.98	11.78	10.23
	P_MAPE_ (%)	26.20	19.55	12.46
EMD-SVR	P_RMSE_ (%)	17.41	10.30	7.56
	P_MAE_ (%)	24.80	12.91	8.52
	P_MAPE_ (%)	45.48	29.81	24.15
EMD-SVR-SP	P_RMSE_ (%)	15.09	8.90	12.43
	P_MAE_ (%)	18.58	12.61	12.84
	P_MAPE_ (%)	40.64	24.08	31.42
EMD-ANN	P_RMSE_ (%)	14.37	5.12	7.71
	P_MAE_ (%)	20.33	6.83	7.72
	P_MAPE_ (%)	27.62	10.22	13.29

1. Compared with the above-mentioned classic individual models, the performance of the EMD-based method is significantly improved. Take wind station #1 as an example, in the 1-step forecasting, the value of RMSE of the EMD-based methods is around 0.60, while the classic individual model is around 1.20. After the wind speed is decomposed by EMD, the value of RMSE is reduced almost doubled.
2. For wind station #1, except for the MAE in the 3-step forecasting, the performance indicators obtained from the proposed model are significantly better than those EMD-based combined models. For the 3-step forecasting, the performance of EMD-SVR and EMD-SVR-SP in MAE is slightly better than the proposed combined model, but in other evaluation indicators, the proposed combined model achieves a significantly better performance. Furthermore, EMD-ANN is always worse in MAPE as compared with the other three combined models, with MAPE of 23.55%, 27.67%, and 29.31% for 1- to 3-step forecasting.
3. For wind station #2, in 1- to 3-step wind speed forecasting, the proposed combined model obtains the best prediction results. The RMSE, MAE and MAPE in the 1-step forecasting are 0.5593, 0.419, and 17.10%, respectively. In comparison, among the other four EMD-based combined models, the EMD-ELM and EMD-ANN models have similar prediction performance in 1- to 3-step forecasting, with MAPE values of 21.59%, 27.49%, 27.65% and 21.83%, 25.3%, 27.86%, respectively.

In total, the EMD-based method has obvious advantages over traditional methods, and the proposed method that using EMD, FS, SVR and LassoCV can achieve better performance.

## Discussion

### Performance of SVR-SP and LassoCV on different IMFs

According to the EMD principle, the frequency of the IMF components is from high to low. The non-linear and non-stationary information of wind speed data is mainly concentrated in the high-frequency IMF, and the low-frequency IMF presents a Sin-like function curve. Based on its characteristics, in this study we use SVR-SP and LassoCV to predict IMFs of different frequencies. In order to verify the effectiveness of this hybrid EMD model, in this section, we take wind station #2 as an example to analyze the performance of the two methods on different IMF components. Table 10 lists the RMSE of SVR-SP and LassoCV on different IMF components. It is worth mentioning that in multi-step prediction, the prediction accuracy of the first step is more important than the other steps, which is of great significance for the accurate estimation of wind power. It can be seen from Table 10 that SVR-SP can obtain significantly better performance than LassoCV at high frequency (IMF1), while LassoCV can obtain better performance at low frequencies (IMF2∼IMF7, Trend), and its RMSE is already close to zero at IMF4. Moreover, SVR-SP has a risk of overfitting when predicting low frequencies, resulting in poor performance. In total, the proposed model that combines the EMD decomposition characteristics and the advantages of the algorithm can achieve better performance than the traditional EMD model.

**Table 10:** The RMSE of SVR-SP and LassoCV on different IMF components at wind station #2.

Steps	Models	IMF1	IMF2	IMF3	IMF4	IMF5	IMF6	IMF7	Trend
1-step	SVR-SP	0.530	0.256	0.061	0.047	0.042	0.040	0.326	0.100
	LassoCV	0.594	0.178	0.033	0.002	0.001	0.000	0.000	0.000
2-step	SVR-SP	0.670	0.407	0.198	0.067	0.041	0.042	0.327	0.100
	LassoCV	0.662	0.369	0.121	0.009	0.001	0.000	0.001	0.000
3-step	SVR-SP	0.668	0.422	0.354	0.086	0.046	0.045	0.327	0.100
	LassoCV	0.663	0.401	0.262	0.023	0.002	0.001	0.001	0.000

### Comparison of different signal decomposition techniques

Besides EMD, Variational Mode Decomposition (VMD) and Ensemble Empirical Mode Decomposition (EEMD) are also widely used in short-term wind speed forecasting. Here, we analyze the impact of different signal decomposition techniques on the performance of our proposed method. Table 11 shows the prediction performance of the three signal decomposition techniques on two wind stations. For wind station #1, it can be found that compared with VMD and EEMD, EMD obtains the best RMSE value in the 1-step forecasting. The performance obtained by VMD in the 1-step and 2-step forecasting is relatively close, but it drops significantly in the 3-step forecasting. EEMD inherits from EMD, similar to EMD, as the step size increases, the performance will decrease significantly. For wind station #2, EMD also obtained the best predictive performance. VMD has a similar conclusion on wind station #1, and the performance of the 1-step and 2-step forecasting is relatively close. It should be pointed out that in multi-step forecasting, the 1-step forecasting is usually used for wind energy estimation, and other steps are used to assist decision-making, so more attention is paid to the performance of the 1-step forecasting.

**Table 11:** The RMSE of VMD, EEMD and EMD at wind stations #1 and #2.

Wind station	Signal decomposition method	RMSE		
		1-step	2-step	3-step
Site #1	VMD	0.6395	0.6782	0.7793
	EEMD	0.6358	0.7301	0.8277
	EMD (The proposed)	0.5859	0.7531	0.8528
Site #2	VMD	0.6664	0.6654	0.7111
	EEMD	0.5844	0.8404	0.8758
	EMD (The proposed)	0.5593	0.7540	0.7911

### The impact of the number of selected features on performance

**Figure 7:**
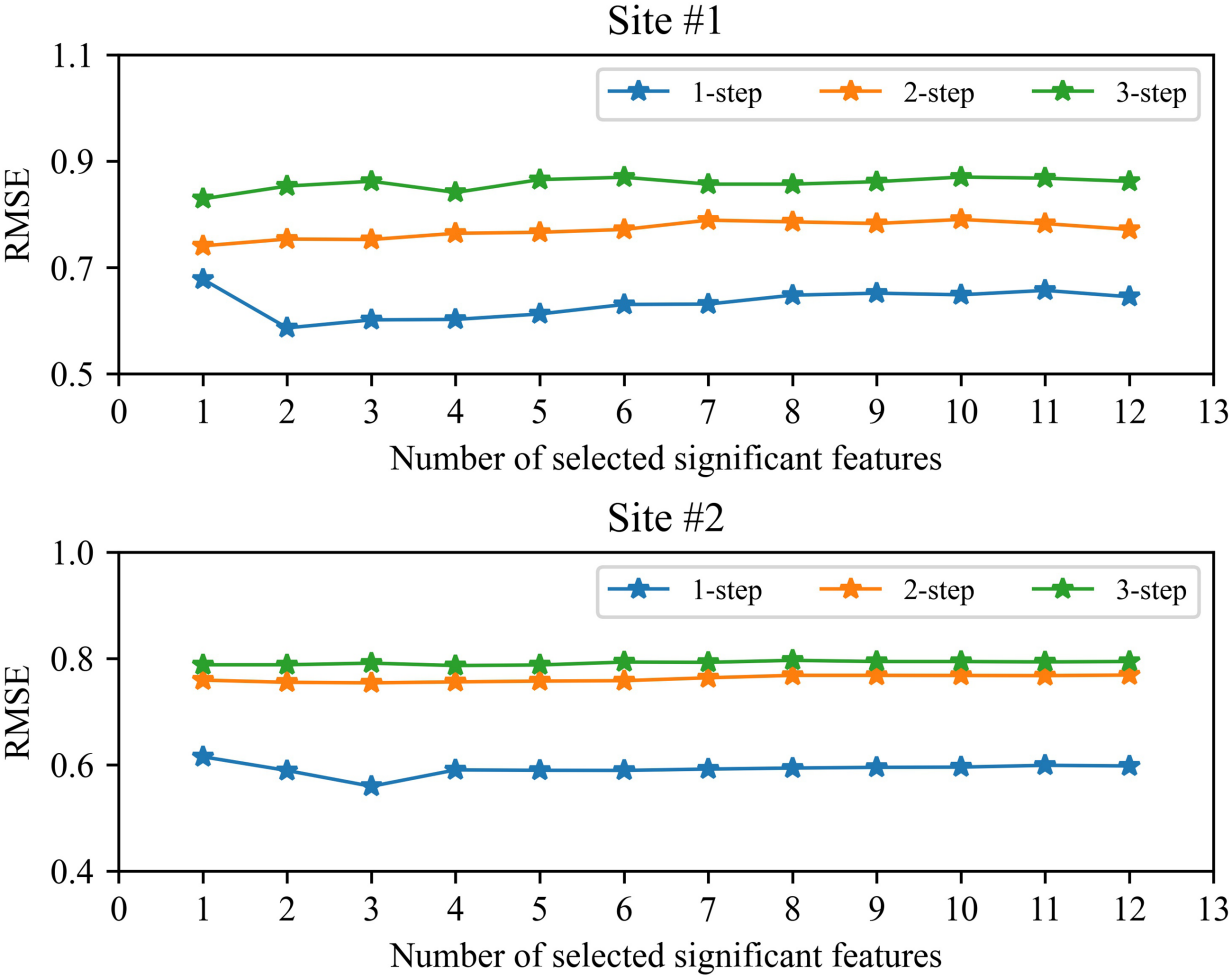
The RMSE between the number of selected features and the performance of the proposed method.

Feature selection is used to remove redundant features in the study. However, the number of selected significant features will more or less affect the short-term wind speed forecasting. In order to ensure the stability in the complicated industrial system, we analyzed the performance of our proposed method under the different number of selected features. Figure 7 shows the RMSE value between the number of selected features and the performance of our proposed method. It should be pointed out that in the study based on the characteristics of EMD decomposition we use FS and SVR to predict high-frequency component (*i.e.,* IMF_1_), and use LassoCV to predict low-frequency components. Feature selection is mainly used in the prediction of IMF_1_ component. From Fig. 7, we can be seen that feature selection can slightly improve the performance of 1-step forecasting, but has little effect on 1-step and 2-step forecasting. Overall, as the number of selected features decreases, the generalization performance of the method will improve, but when the selected features are too scarce, the performance will drop sharply due to the deletion of useful features. In order to determine the appropriate number of features, by following (Bradley, Mangasarian & Street, 1998; Chizi, Rokach & Maimon, 2009) , this study uses cross-validation to select.

### Performance under different signal-to-noise ratios

**Figure 8:**
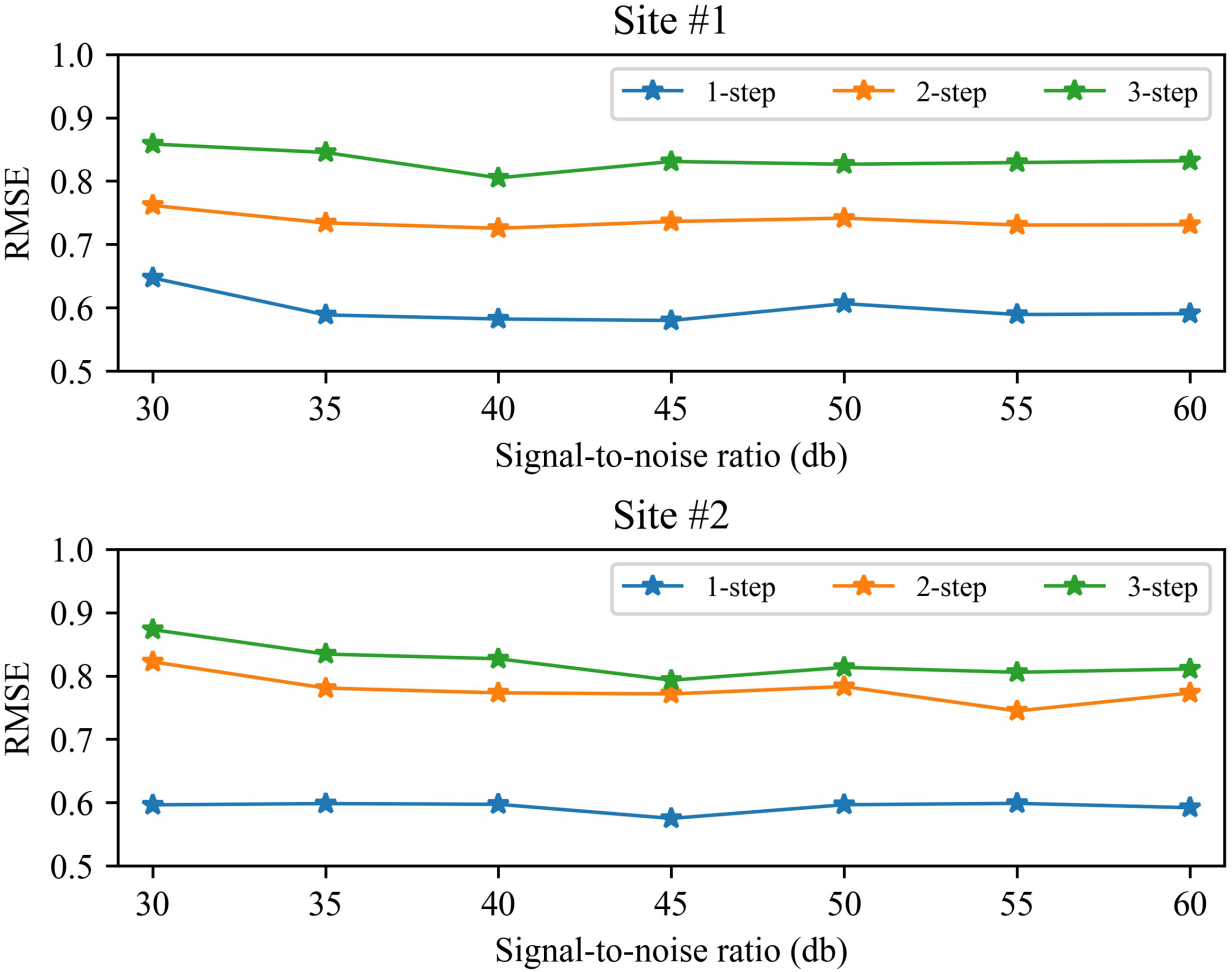
The RMSE of the proposed method at 30 60db SNR.

In the process of collecting wind speed, it is often affected by the environment and the anemometer itself, resulting in a certain amount of noise in the data. In order to verify the reliability of the method, we analyzed the prediction performance under different signal-to-noise ratios (SNRs). Figure 8 shows the 1-step to 3-step prediction performance of the method from 30∼60db SNR. Take wind station #1 as an example, it can be seen from Fig. 8 that the performance of the proposed method is relatively stable under different signal-to-noise ratios. The RMSE value of 1-step forecasting is about 0.6, the RMSE value of 2-step forecasting is about 0.75, and the RMSE value of 3-step forecasting is about 0.85. In general, as the signal-to-noise ratio increases, the prediction performance of the proposed method will be improved. Similar performance also exists on site #2. These experimental results show that the proposed method can accurately predict wind speed under certain noise.

## Conclusions

As a sustainable and renewable energy, wind power has attracted widespread attention and rapid development in recent years. Reliable and accurate wind speed forecasting will provide support for wind power planning and control. Due to the non-linearity and non-stationarity of wind, forecasting is still a difficult yet challenging problem. In the paper, we developed a new wind speed forecasting model based on EMD, FS, SVR and LassoCV. EMD is employed to extract IMFs from the original non-stationary wind speed time series. FS and SVR are combined to predict the high-frequency IMF. LassoCV is adopted to complete the prediction of low-frequency IMF and trend. By testing in two wind speeds obtained from Michigan, USA, the experimental results show that under 1- to 3-step forecasting the proposed model can achieve better prediction performance than the classic individual and traditional EMD combined models. Although the proposed model has achieved good performance, it still has some limitations. After the new data is updated, the model needs to be retrained. In future research, we will try to integrate online learning in our proposed method.

## Additional Information and Declarations

### Data Availability

The following information was supplied regarding data availability: The code is available at GitHub: https://github.com/JackAndCole/short-term-wind-speed-forecasting. The data is available at GitHub: https://github.com/JackAndCole/short-term-wind-speed-forecasting/tree/main/data.
